# Transcriptional consequences of *MBD5* disruption in mouse brain and CRISPR-derived neurons

**DOI:** 10.1186/s13229-020-00354-1

**Published:** 2020-06-05

**Authors:** Catarina M. Seabra, Tatsiana Aneichyk, Serkan Erdin, Derek J. C. Tai, Celine E. F. De Esch, Parisa Razaz, Yu An, Poornima Manavalan, Ashok Ragavendran, Alexei Stortchevoi, Clemer Abad, Juan I. Young, Patricia Maciel, Michael E. Talkowski, James F. Gusella

**Affiliations:** 1 Molecular Neurogenetics Unit, Center for Genomic Medicine, Massachusetts General Hospital, Boston, MA USA; 2 Program in Medical and Population Genetics, Broad Institute of MIT and Harvard Medical School, Boston, MA USA; 3 GABBA Program - Institute of Biomedical Sciences Abel Salazar of the University of Porto, Porto, Portugal; 4 Independent Data Lab UG, Munich, Germany; 5 Department of Neurology, Harvard Medical School, Boston, MA USA; 6 Human Phenome Institute, Fudan University, Shanghai, China; 7 Center for Computational Biology of Human Disease & Center for Computation and Visualization, Brown University, Providence, Rhode Island USA; 8 Dr. John T. Macdonald Foundation Department of Human Genetics, University of Miami, Miami, FL USA; 9 Life and Health Sciences Research Institute (ICVS), School of Medicine, University of Minho, Braga, Portugal; 10 ICVS/3B’s - PT Government Associate Laboratory, Braga, Guimarães Portugal; 11 Department of Genetics, Blavatnik Institute, Harvard Medical School, Boston, MA USA; 12 Harvard Stem Cell Institute, Harvard University, Cambridge, MA USA

**Keywords:** MBD5, Autism spectrum disorder, NDD, CRISPR, Mouse, Neurons, Transcriptomics

## Abstract

**Background:**

*MBD5*, encoding the methyl-CpG-binding domain 5 protein, has been proposed as a necessary and sufficient driver of the 2q23.1 microdeletion syndrome. De novo missense and protein-truncating variants from exome sequencing studies have directly implicated *MBD5* in the etiology of autism spectrum disorder (ASD) and related neurodevelopmental disorders (NDDs). However, little is known concerning the specific function(s) of MBD5.

**Methods:**

To gain insight into the complex interactions associated with alteration of *MBD5* in individuals with ASD and related NDDs, we explored the transcriptional landscape of *MBD5* haploinsufficiency across multiple mouse brain regions of a heterozygous hypomorphic *Mbd5*^+/GT^ mouse model, and compared these results to CRISPR-mediated mutations of *MBD5* in human iPSC-derived neuronal models.

**Results:**

Gene expression analyses across three brain regions from *Mbd5*^+/GT^ mice showed subtle transcriptional changes, with cortex displaying the most widespread changes following *Mbd5* reduction, indicating context-dependent effects. Comparison with *MBD5* reduction in human neuronal cells reinforced the context-dependence of gene expression changes due to MBD5 deficiency. Gene co-expression network analyses revealed gene clusters that were associated with reduced *MBD5* expression and enriched for terms related to ciliary function.

**Limitations:**

These analyses included a limited number of mouse brain regions and neuronal models, and the effects of the gene knockdown are subtle. As such, these results will not reflect the full extent of *MBD5* disruption across human brain regions during early neurodevelopment in ASD, or capture the diverse spectrum of cell-type-specific changes associated with *MBD5* alterations.

**Conclusions:**

Our study points to modest and context-dependent transcriptional consequences of *Mbd5* disruption in the brain. It also suggests a possible link between *MBD5* and perturbations in ciliary function, which is an established pathogenic mechanism in developmental disorders and syndromes.

## Background

The 2q23.1 microdeletion syndrome is a genomic disorder characterized by intellectual disability (ID), severe speech impairment, seizures, behavioral problems, microcephaly, mild craniofacial dysmorphism, small hands and feet, short stature, and broad-based ataxic gait [1–4]. *MBD5* (OMIM 611472), encoding the methyl-CpG-binding domain 5 protein, has been implicated as the driver of 2q23.1 microdeletion syndrome [5], while subsequent studies have characterized phenotypes associated with reciprocal dosage change [6, 7]. In addition to large copy number variants (CNVs), de novo missense and protein-truncating variants from exome sequencing studies have also directly implicated *MBD5* in the etiology of autism spectrum disorder (ASD) and related neurodevelopmental disorders (NDDs) [8–10].

The MBD5 protein belongs to the methyl-CpG-binding domain (MBD) family, which includes *MBD1* - *MBD6*, *SETDB1*, *SETDB2*, and *MECP2*, the causative gene for Rett syndrome. The MBD family members have key roles in regulating gene transcription, and in vitro experiments have led to a model in which MBD1, MBD2, MBD4, and MECP2 recruit chromatin remodelers, histone deacetylases, and methylases to methylated DNA, leading to transcriptional repression [11, 12]. Indeed, their MBD allows the specific recognition of DNA containing methylated cytosine and, as a consequence, the proteins serve as interpreters of DNA methylation [13]. The MBD family members are also involved in a variety of functions including DNA damage repair (MBD4), histone methylation (SETDB1 and SETDB2), and X chromosome inactivation (MBD2) [14, 15], transcript splicing and gene activation [16–18]. However, little is known concerning the specific function(s) of MBD5. Immunocytochemistry experiments have shown that MBD5 localizes in the nucleus to non-heterochromatin regions, which suggests that it acts as a transcriptional activator [19]. Notably, it does not have the ability to bind methylated DNA in vitro [13].

**Fig. 1:**
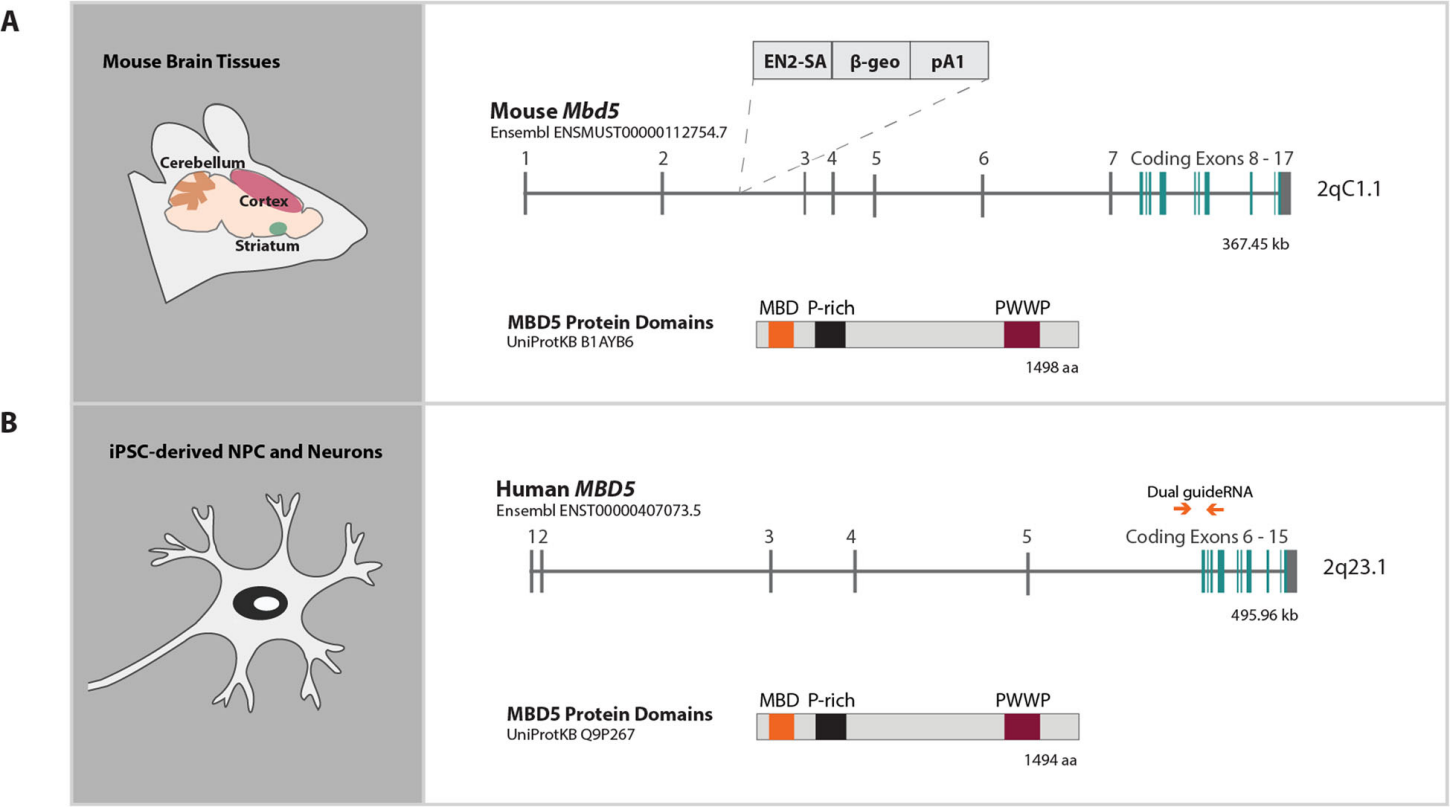
Study design and comparison of mouse *Mbd5 and* human *MBD5* gene and protein structure. **a** Left—representation of the mouse brain regions of the *Mbd5*^*+/GT*^ mouse model^14^ analyzed (cerebellum, cortex, and striatum). Right—schematic representation of the mouse *Mbd5* gene found on 2qC1.1, including the gene-trap cassette inserted within intron 2^14^. Below, the canonical protein isoform 1, the main described isoform, composed of a conserved MBD, a proline-rich segment (*P* rich), a PWWP domain. **b** Left—human iPSC-derived NPCs and neurons were used to generate isogenic edited cell lines. Right—schematic representation of the human *MBD5* gene structure found on 2q23.1, including the dual guideRNA strategy for CRISPR/Cas9 editing targeting exon 6. Below, protein isoform 1, the most expressed in brain

The human *MBD5* gene is composed of 15 exons, but only exons 6 to 15 represent coding sequence. The translation of the canonical transcript MBD5-001 (ENST00000407073) leads to the production of the main protein isoform reported to date, isoform 1 (UniProtKB ID Q9P267), which comprises 1494 amino acids (Fig. 1b). Isoform 1 contains two conserved domains, a methyl binding domain (MBD) and a proline and tryptophan-rich domain (PWWP), both of which may be found in chromatin-associated proteins involved in transcriptional regulation. Notably, alterations to genes within these pathways involving transcriptional regulation have been reproducibly implicated in ASD and more broadly defined NDDs [8, 10, 20–23], and multiple studies have also suggested protein-protein interactions (PPI) between MBD5 and the products of other NDD associated genes, including interaction with KDM1B within the PR-DUB polycomb protein complex [24], as well as regulatory interactions between *MBD5* transcripts and FMRP (*FMR1*) [25], although the mechanisms by which such interactions contribute to abnormal neurodevelopment are not well understood.

The mouse *Mbd5* gene spans 17 exons with the coding sequence limited to exons 8 through 17. The human and mouse proteins show a 95.18% identity. Expression studies in mouse have shown that MBD5 is expressed in all tissues but most highly in brain and testis [8]. Multiple mouse models have been generated to alter or abolish *Mbd5* expression, including an *Mbd5* knockout (*Mbd5*^*−*/−^), a brain-specific *Mbd5* conditional knockout (*Mbd5*^*f*/−^, NestinCre) [26], and a heterozygous hypomorph (*Mbd5*^+/GT^) [19]. The brain-specific conditional knockout of *Mbd5* results in phenotypes similar to those of constitutive deletion, indicating its crucial role within the nervous system [26]. The homozygous *Mbd5*^GT/GT^ mice exhibit perinatal lethality while heterozygous *Mbd5*^+/GT^ mice are viable [19]. This murine model carries an insertional mutation in *Mbd5* intron 2*,* generated through gene-trap mutagenesis (Fig. 1a). *Mbd5*^+/GT^ mice display some characteristics that may be related to the cardinal phenotypes of 2q23.1 microdeletion carriers, including abnormal social behavior, cognitive impairment, and motor and craniofacial abnormalities (abnormal nasal bone). They are notably small, with reduced body weight and neuromuscular strength, and show motor deficits. Additionally, neuronal cultures from *Mbd5*^+/GT^ mice revealed a deficiency in neurite outgrowth.

To gain insight into the molecular consequences associated with genetic alteration of *MBD5*, we explored the transcriptional landscape of *Mbd5*^+/GT^ and wild-type (WT) mice across multiple brain regions and compared these results to isogenic human iPSC (induced pluripotent stem cell)-derived neuronal cells with CRISPR-mediated mutations of *MBD5*. Our experimental design sought to identify altered genes and pathways relevant to NDD that were associated with partial loss of MBD5 across multiple brain regions. These analyses revealed that *MBD5* disruption leads subtle effects on gene expression that are highly context-dependent, suggesting that MBD5’s role is not regulation of a fixed set of pathways across all cell types but rather that it participates in regulation of genes in a cell type- and potentially stage-specific manner.

## Methods

### *Mbd5*^+/GT^ mouse model

A C57BL/6 background mouse model carrying an insertional mutation in the *Mbd5* locus (B6;CB-Mbd5Gt(pU-21B)205Imeg) was previously generated at the Institute of Resource Development and Analysis (IRDA), Kumamoto University, using a gene-trap construct pU-21B that randomly inserted into the second intron in embryonic stem cells from the line Ayu21-B205. The insertional mutation creates a hypomorphic allele with (Fig. 1a) *Mbd5* expression reduced sufficiently to produce neurodevelopmental abnormalities in heterozygous mice and perinatal lethality in homozygous mice [19]. For this study, we used 10 *Mbd5*^+/GT^ mice and 8 wild-type mice at 8 weeks of age. Three brain regions were collected from each animal—cortex, striatum, and cerebellum—and RNA from each region was extracted separately. Mouse information is detailed in Supplementary Table 1.

### Generation of CRISPR/Cas9-edited iPSC lines

To further explore the relevance of the mouse results in human cells, we derived human neuronal models of *MBD5* disruption from CRISPR/Cas9 gene-edited iPSCs (8330-8) reprogrammed previously from fibroblasts of a healthy male individual using standard retroviral vectors [27]. Pluripotency was confirmed by differentiating into all three embryonic germ layers (Fig. 4a). Dual-guide RNAs were designed based on genome assembly GRCh37 to delete exon 6 of *MBD5*, which is the first coding exon. To assure specificity, multiple online tools were employed: (i) CRISPR design tool (http://tools.genome-engineering.org/) which takes into account off-target predicted sites and gives a score inversely correlated with the number of off-target matches; (ii) sgRNA Designer (http://www.broadinstitute.org/rnai/public/analysis-tools/sgrna-design) which accounts for on-target efficiency [28], and (iii) BLAST (NCBI) to query for the guide sequences, including the PAM motif, to determine whether the sequences target uniquely to the desired regions or if there are any potential off-target sites.

The iPSCs (1 × 10^6^ cells) were transfected with 1 μg total DNA plasmid, Cas9 expression vector pX459 (pSpCas9(BB)-2A-Puro - Addgene plasmid 48139) along with the chosen gRNAs (inserted into pGuide - Addgene plasmid 41824) and an external EGFP (enhanced green fluorescent protein) vector. For nucleofection of the gRNAs into the iPSC, the Human Stem Cell Nucleofector Kit 1 (Lonza) and Amaxa Nucleofection II device (Lonza) were used with program B-016, according to the manufacturer’s instructions. After nucleofection, iPSCs were cultured on Matrigel-coated wells using conditioned mTeSR medium (StemCell Technologies) supplemented with 10 μM ROCK inhibitor (Y-27632 dihydrochloride, Santa Cruz Biotech) and 10 ng/ml bFGF (R&D).

Three *MBD5*-edited cell lines with alterations in the conserved MBD domain were used in this study (Fig. 4c). Cell line 1 is a compound heterozygote cell line (*6het AIVG12*), generated using guideRNAs 6.8 and 6.5 (Supplementary Table 2)*.* The first allele contains a 4 bp deletion (NM_018328.4 (*MBD5*): c.66_70del) that is predicted to lead to a prematurely truncated peptide of only 80 amino acids. The second allele bears a 4 bp deletion (NM_018328.4(*MBD5*):c.66_70del) followed by a 16 bp insertion (NM_018328.4(*MBD5*):c.70_86ins) that results in an in-frame insertion of four amino acids (Q9P267.3(MBD5):p.V23_G24insYTSS) and a substitution of one amino acid in the canonical protein isoform (Q9P267.3 (MBD5):p.G24C), predicting a 1498 amino acid protein from this allele. Cell lines 2 and 3 (*6het AIIIB5* and *6hom AIID2)* harbored heterozygous and homozygous alterations, respectively, which were formed by non-homologous end joining (NHEJ) that resulted in precise editing at the genomic target sites without further changes by the guideRNAs 6.3 and 6.8 (Supplementary Table 2). Gene editing in cell lines 2 and 3 led to the precise excision of 41 bp within exon 6 (NM_018328.4(*MBD5*): c.23_64del), removing the initial portion of the conserved MBD domain. As this modification causes a frameshift of the open reading frame, it is predicted to result in a prematurely truncated peptide of 38 amino acids, considering the canonical MBD5 protein isoform. MBD5 protein levels could not be assessed as no suitable antibodies were found upon stringent testing of commercially available reagents. Three cell lines were used as controls, namely (a) a wild-type ( “non-treated”) 8330-8 iPSC line; (b) an 8330-8 iPSC line transfected only with Cas9 (“Cas9 only”); (c) a negative control that resembles a wild-type cell line, which was transfected with CRISPR/Cas9 however no cuts in the targeted sites were obtained.

### iPSC-derived neuronal differentiation

Expandable neuronal progenitor cells (NPCs) were generated from iPSCs through differentiation by the embryoid body (EB) protocol using STEMdiff™ Neural Induction Medium (StemCell Technologies), following the manufacturer’s instructions. Briefly, 3 × 10^6^ iPSCs were transferred to a micro-patterned culture surface well (AggreWell™800) using centrifugal force, resulting in 10,000 cells per micro-well that would then form EBs (day 0). EBs exhibited the typical spherical and well-limited appearance of EBs formed from embryonic stem cells. EBs were plated on day 5 onto Corning® Matrigel®-coated plates and cultured for the following days. Around day 12, neural rosette structures, mimicking the apicobasal organization of the neural tube epithelium, were visible and were manually collected using DMEM-F12 medium and collected into a 15-mL tube and plated onto poly-ornithine (PLO, Sigma)/laminin (Sigma) coated culture plates, at a final concentration of 20 μg/mL and laminin at 5 μg/mL. These cells were then expanded in NPC expansion medium containing 70% DMEM (Invitrogen), 30% Ham’s F-12 (Mediatech) supplemented with 2% B-27 (Invitrogen), 1% Pen/Strep/Glutamine (Corning), heparin (5 μg/mL, Sigma) and mitogens EGF (20 ng/mL, Sigma), and bFGF (20 ng/mL, R&D Systems) (Fig. 4b).

After 10 passages, NPCs were used and analyzed for expression of NPC-specific markers. Immunofluorescence staining was performed after fixation in 4% paraformaldehyde, followed by primary antibody incubation with rabbit anti-human NESTIN (1:500 dilution, Millipore ABD69), mouse anti-human SOX1 (1:200 dilution, Millipore AB15766), rabbit anti-SOX2 (1:200 dilution, Abcam AB59776), and rabbit anti-human PAX6 (1:200 dilution Covance PRB278P) and subsequent appropriate fluorochrome-conjugated secondary antibodies (1:400 dilution) for microscopic evaluation (Fig. 4d).

Terminal neuronal differentiation was achieved by plating expanded NPCs at a seeding density of 2 × 10^6^ cells per well on polyornithine/laminin-coated plates (coated together overnight) in NPC expansion medium lacking both growth factors EGF and bFGF and heparin, with medium replacement every 3–5 days for 30 days. Neuronal-specific markers were assessed in mature neurons, using chicken anti-human MAP2 (1:2500 dilution, EnCor Biotechnology Inc CPCA-MAP2), and mouse anti-human SMI312 (1:1000 dilution, Biolegend 837901) (Fig. 4e). Fluorescence intensity was normalized to the 8330-8 non-treated control sample.

The presence of NPC-specific markers NESTIN, PAX6 and SOX1 and SOX2 and neuron-specific markers MAP2 and SMI312 in the differentiated cell lines indicates effective differentiation, with the exception of the compound heterozygote cell line *6het AIVG12* which did not fully complete the differentiation process, indicating compromised neuronal development.

### RNA extraction and library preparation

RNA from cell lines was obtained by lysing 1-2 million cells using 1 mL of Trizol (Invitrogen) then mixed with 1/5th volume of chloroform and centrifuged at 200×*g* for 5 min. The aqueous phase was collected and processed using a RNeasy Mini column (Qiagen). cDNA was synthesized from 1 μg of extracted RNA using SuperScript® III Reverse Transcriptase (ThermoFisher Scientific) with oligo(dT), random hexamers, and RNase inhibitor. Mouse brain tissue samples were collected from the cortex, cerebellum and striatum of heterozygous *Mbd5*^+/GT^ mice and wild-type controls at 8 weeks. Tissues that were previously frozen at −80°C were thawed overnight at −20°C submerged in RNAlater®-ICE Frozen Tissue Transition Solution (ThermoFisher Scientific) enabling easy cutting and extraction of high-quality RNA. RNA from tissues was obtained by lysing in 1 mL of Trizol (Invitrogen) using metallic pellets (Qiagen) and a tissue lyser, then mixed with 1/5th volume of chloroform and centrifuged at 200×*g* for 5 min. The aqueous phase was collected and mixed with isopropanol and centrifuged to obtain a pellet that was then washed with 75% ethanol and air dried and resuspended in RNAse-free water. Each tissue type was collected on the same day to avoid batch effects. The RNAseq library was prepared as previously described [29].

### Computational methods

Quality control (QC) assessment of sequence reads was performed using fastQC (v. 0.10.1 http://www.bioinformatics.babraham.ac.uk/projects/fastqc/), and reads were aligned to human reference genome Ensembl GRCh37 (v. 75) and to mouse reference genome Ensembl GRCm38 (v. 83) for cell lines and mouse brain regions respectively using STAR (v. 2.4.2) with its default parameter settings, followed by QC with Picard Tools (http://broadinstitute.github.io/picard/), SamTools [30], and MultiQC. Counts were generated using STAR aligner option ‘--quantMode GeneCounts’ for all Ensembl genes (GRCh37 v.75; GRCm38 v. 83).

Differential expression analyses included all genes with greater than 4 counts in as many samples as the smallest genotype group. For each mouse brain region and each cell type, counts were first normalized using DESeq2 [31] function counts with option normalized = TRUE. Surrogate variable analysis (SVA), implemented in R package sva [32], was used to identify variables that influence gene expression profiles, and such variables were added to the design matrix (together with genotype) during analysis of differentially expressed genes (DEGs). SVA was performed using model matrix ~ Genotype and null matrix ~1. In the cerebellum, two surrogate variables were identified, while four variables were found in the striatum and cortex, respectively. In cell lines, two surrogate variables were identified in NPCs and in neurons. *P* values for enrichments for DEGs in compilation of gene lists relevant for neurodevelopment was performed using Fisher’s test. Enrichments were considered significant if *p* value < 0.05×(number of gene lists tested), which would be equivalent to Bonferroni-corrected *p* value < 0.05.

Co-expression network analysis was performed using R package WGCNA [33]. WGCNA was applied to the genes with counts > 10 in at least half of the samples. Counts were normalized using variance stabilizing transformation implemented by the corresponding function in R package DEseq2. The minimum module size was set to 10, and the dissimilarity threshold for module merging was set to 0.25. Soft power was selected such that R^2^ for topology free structure model was > 0.8 (cerebellum = 2, cortex = 14, striatum = 17). Co-expression modules were detected using WGCNA function blockwiseModules with parameters corType = ‘bicor’, maxPOutliers = 0.10. All the networks were signed. Module enrichments for DEG and other gene lists were performed using Fisher’s test and adjusted for multiple hypothesis testing using Bonferroni adjustment (adjusting for total number of performed tests per brain region = number of gene lists including DEG lists×(number of modules). Gene ontology enrichments were performed using topGO R package with Fisher’s test and algorithm “weight01” to take into account GO tree structure. *P* value adjustment was performed using Bonferroni correction method.

For meta-analysis, Fisher’s method for combining *p* values was applied. As Fisher’s method does not incorporate information about directionality, we sub-selected only the genes that have estimated log2 fold changes in the same direction in all comparisons. We then applied Fisher’s method to the *p* values and performed an adjustment for multiple hypothesis testing on the meta *p* values using the Benjamini-Hochberg method.

## Results

### Impact of *Mbd5* disruption across brain regions

**Fig. 2:**
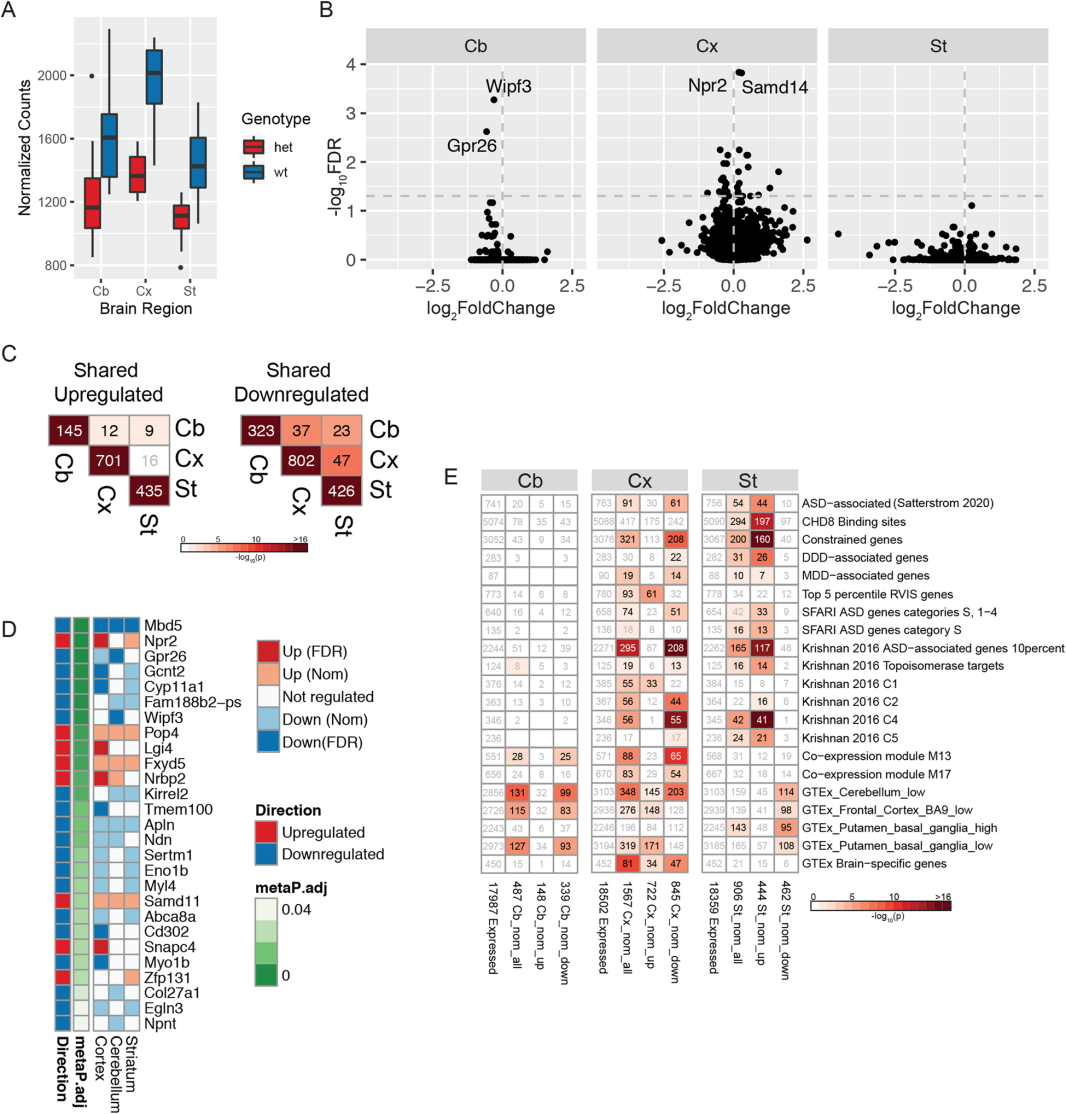
Differential expression analysis of RNA-seq data from mouse brain regions. **a** Expression of *Mbd5* mRNA as normalized counts in three brain regions: cerebellum (Cb), striatum (St), and cortex (Cx). **b** Volcano plots of differential expression analysis per region. **c** Gene overlaps in differentially expressed genes in mouse brain regions. The color indicates the significance (−log_10_(p)) of Fisher’s enrichment test, and the number shows the number of overlapping DEGs at nominal significance. Only the genes that were present in all tests were considered as background for enrichment tests and for overlap analyses. **d** Meta-analysis of *p* values from differential gene expression comparisons in each brain region using Fisher’s method. The color in the heatmap shows whether the gene was significantly up- or downregulated in a corresponding brain region. “Direction” indicates whether the gene was analyzed in a group of upregulated genes or downregulated genes. “metaP.adj” shows the *p* value of meta-analysis using Fisher’s method. **e** Enrichments of differentially expressed genes at FDR and at nominal significance in gene sets previously associated with brain development and developmental disorders. The description of gene lists is provided in Supplementary Tables 5 & 6.

We analyzed genome-wide expression differences between ten *Mbd5*^+/GT^ and eight matched wild-type mice at 8 weeks of age in three distinct brain regions: cerebellum, striatum, and cortex. *Mbd5* expression was reduced in all three tissues, with a slightly more moderate effect in the cerebellum (log_2_FC = −0.35, FDR = 2.2 × 10^−6^) than in striatum (log_2_FC = −0.51, FDR = 3.7 × 10^−20^) and cortex (log_2_FC = −0.46, FDR = 4.2 × 10^−17^) (Fig. 2a). The number of genes differentially expressed at FDR < 0.05 in our model was low, with the greatest differences in cortex (35 genes, 16 displaying increased expression and 19 reduced expression) (Fig. 2b and Supplementary Table 3). Cerebellum showed only downregulation of two genes in addition to *Mbd5* - *Wipf3* and *Gpr26*, while striatum, in which the reduced *Mbd5* expression was most significant, exhibited no other differentially expressed genes at FDR < 0.05. Even among nominally significant DEGs (nDEGs *p* < 0.05; cortex *n* = 1567; striatum *n* = 906; cerebellum *n* = 487), there was limited overlap, with significant similarities being seen only among downregulated genes (Fig. 2c and Supplementary Table 4), indicating that the effects of *Mbd5* reduction are largely different across these three brain regions. This conclusion was reinforced when we performed a meta-analysis across the mouse brain regions as a potential route to identify genes consistently dysregulated. From 2123 genes with positive log2 fold changes in all three regions and 2961 genes with negative log2 fold changes in all three, we obtained only 27 significant genes: 8 upregulated and 19 downregulated (Fig. 2d). However, even among these genes, only 5 were nDEGs in all three brain regions, including, as expected, *Mbd5* as the most significantly dysregulated gene. Thus, while *Mbd5* reduced expression was readily detected in all three brain regions, its effects were largely distinct across cortex, cerebellum, and striatum.

We next investigated whether these DEGs were enriched for genes associated with ASD and/or NDDs, first using the limited set of DEGs at FDR < 0.05 and then the larger, less stringently defined set of nDEGs. We tested over 100 relevant gene lists, many of which were highly correlated with each other, including: genes within known common autism-associated deletion/duplication regions, genes from a study of NDD families (DDD: deciphering developmental disorders) identified to have loss-of-function or missense mutations, the genotype tissue expression project (GTEx)-derived brain-specific genes and genes specific for various brain regions, ASD-associated genes curated from the Simons Foundation for Autism Research Initiative (SFARI), genes from BrainSpan brain development co-expression modules and networks as described in Troyanskaya et al., and genes associated with ASD from a broad swath of studies including de novo mutations and/or applying a Bayesian Transmission and de novo Association framework (TADA) [8, 10, 20, 22, 24, 34–59]. Significant enrichments are shown in Fig. 2e. Cortex downregulated nDEGs were enriched for brain specific and constrained genes (i.e., genes exhibiting fewer loss-of-function variants than expected in population-based samples) [60, 61] and in developmental brain co-expression modules from BrainSpan data [62], as well as gene clusters described in Krishnan et al. [34] associated with histone modification (C4) and embryonic development and morphogenesis (C2). The specific BrainSpan co-expression modules enriched in nDEGs (modules M13 and M17 in Parikshak et al.; Fig. 2e) have been associated with synaptic plasticity and are characterized by increased expression during late fetal and early neonatal development [38]. In striatum, we observed enrichment of upregulated nDEGs for constrained genes as well as CHD8 binding sites, ASD-associated genes and several co-expression modules described by Krishnan et al. [34]. A complete list of tested gene lists and their descriptions can be found in Supplementary Table 5 and a delineation of all genes in these lists in Supplementary Table 6. A heatmap of all *p* values and overlap counts is provided in Supplementary Figure 1.

### Co-expression network analyses suggest regulation of transcriptional modules

**Fig. 3:**
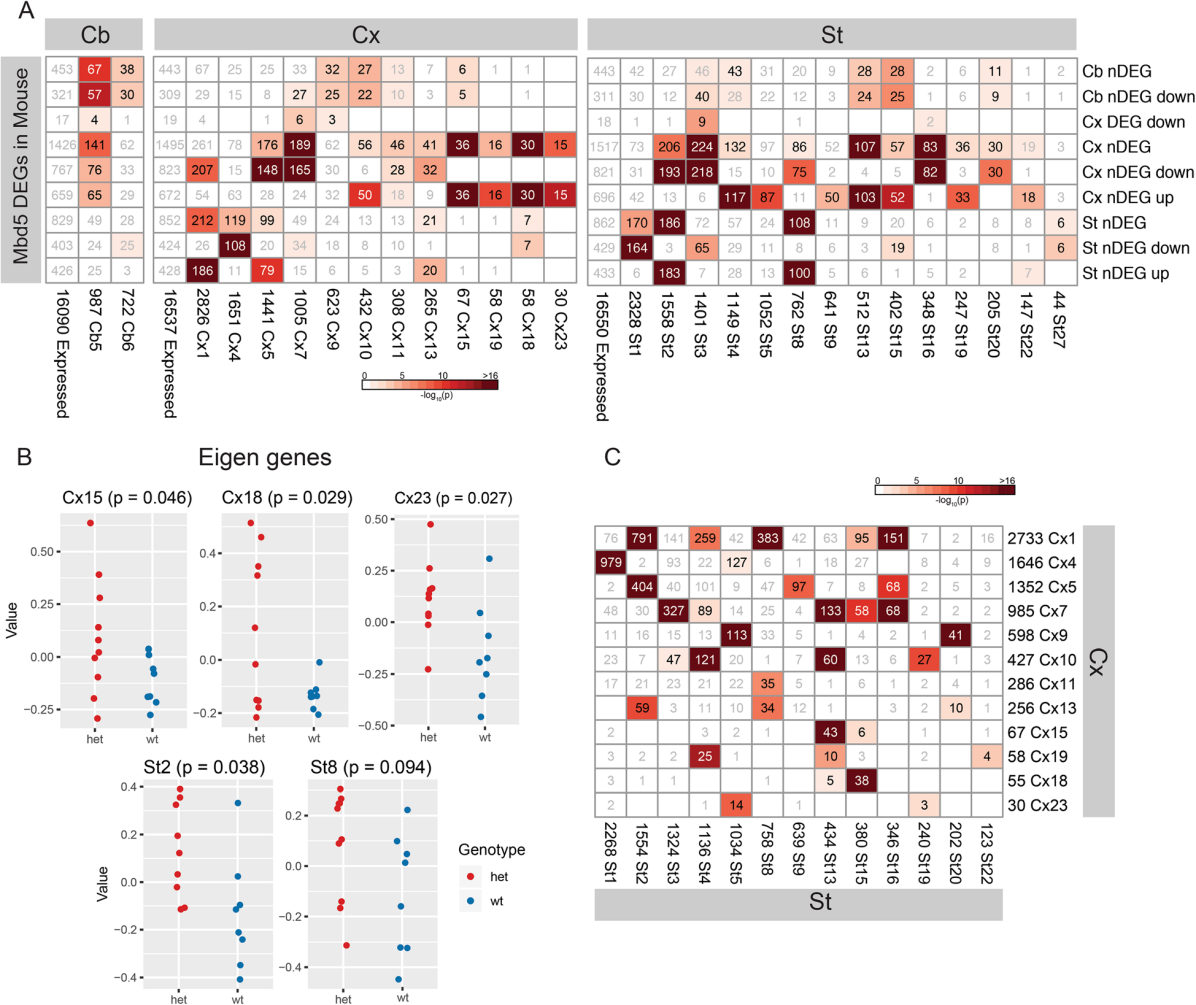
Co-expression network analysis in mouse brain regions. **a** Enrichments of co-expression modules in each brain region for differentially expressed genes. Only modules with significant enrichments are shown. The color of the heatmap shows the −log10(*p*) of Fisher’s enrichment test, and the number shows number of genes that overlap. The number next to the module name in the columns shows total number of expressed genes in that module. **b** Eigenvalues of modules with significant differences in values between two genotypes. The value in each plot title shows corresponding *p* value of *t* test between eigenvalues of two genotypes. **c** Enrichments of genes from striatum and cortex co-expression modules. Only modules with enrichment for nDEGs and a corresponding overlapping module from the other brain region are shown. The color of the heatmap shows the −log10(*p*) of Fisher’s enrichment test, and the number shows number of genes that overlap. The number next to the module name in the columns shows total number of expressed genes in that module

Weighted gene co-expression network analysis (WGCNA) can facilitate discovery of biological processes and functions that are co-regulated at the transcriptional level in response to a genomic perturbation. Often, such analyses will identify modules with a large number of genes that are involved in fundamental cellular processes; however, they can also identify co-expression of clusters of genes that are demonstrably correlated with the genotype or phenotypic feature. We performed WGCNA across all expressed genes in each brain region to investigate whether DEGs formed a separate single co-expression module or were distributed across several distinct clusters. The number of co-expression modules varied across brain regions, with 11, 25, and 37 modules in the cerebellum, cortex, and striatum, respectively. We tested each module for nDEGs as well as for the pre-defined gene sets of relevance to NDD described above (Fig. 3a, Supplementary Figure 2, Supplementary Tables 5 & 6). In addition, in order to identify the modules with the highest relevance to *Mbd5* effects, we tested eigenvalues of the eigen genes of each module for differences in genotype using a t-test (Fig. 3b).

In the cerebellum, most nDEGs appeared in the modules Cb5 and Cb6; however, the eigenvalues of these modules did not show any differences between genotypes. In cortex and striatum, 11 and 5 modules correspondingly showed enrichments for nDEGs. However, only 3 modules in cortex (Cx15, Cx18, and Cx23) and 2 modules in striatum (St2 and St8) showed significant genotype differences in eigenvalues (*t* test *p* value < 0.1). Module Cx15 was enriched for the GO term “cilium movement” (pBonf = 4.8 × 10^−5^). Supplementary Figure 3 depicts module Cx15 which is enriched for upregulated nDEGs from cortex (circled in red). Those nodes that are red in color are the genes associated with cilia. Another distinctive feature of this module is that it consists of genes with relatively low expression in the brain (median normalized counts = 47). Taken together these features suggest the possibility that an early effect of Mbd5 on cilia development and function in these mice acts via genes which later, at 8 weeks, show upregulation of what is normally a low expression level at this age. Module Cx18 showed an enrichment for GO terms “extracellular space” (pBonf = 0.002) and “collagen trimer” (pBonf = 0.038). In striatum module St2 GO terms “cytoplasmic stress granule” (pBonf = 0.022) and “DNA binding” (pBonf = 0.0002) showed significant enrichment. Modules Cx23 and St8 did not show enrichment for any GO terms.

Several modules in striatum (e.g., St4, St13, St16) displayed intriguing enrichment for cortical nDEGs, but not for striatal nDEGs (Fig. 3a) suggesting that they reflect processes common to both brain regions that are dysregulated only in cortex by *Mbd5* reduction. Similarly, Cx4 showed enrichment for nDEGs from striatum, but not nDEGs from cortex. Other modules showed evidence for enrichment of nDEGs dysregulated in opposite directions in cortex and striatum (e.g., Cx1, Cx5, St2, St8), suggesting different effects of Mbd5 on the same modules in different brain regions. We therefore tested the modules in striatum and cortex for correspondence and were able to establish matching modules in both brain regions (Fig. 3c). Most notably, Cx15, the module associated with ciliary function in cortex was correlated with St13 in striatum, which also revealed an association with cilium (GO term “motile cilium” pBonf = 9.9 × 10^−9^). Both St1 and Cx4 modules showed dramatic enrichments for numerous terms associated with translation and with mitochondria (with *p* values < 10^−10^). Similarly, Cx18 and St15 both show enrichments for the same terms: “extracellular space” (pBonf = 1.7 × 10^−18^) and “collagen trimer” (pBonf = 0.0014). Taken together, the WGCNA module and nDEG enrichment analyses suggest that corresponding biological processes are active in both striatum and cortex, but that these processes may be affected differently (or in some cases not at all) by *Mbd5* deletion in different brain regions.

### Overlap between cell lines and mouse

**Fig. 4:**
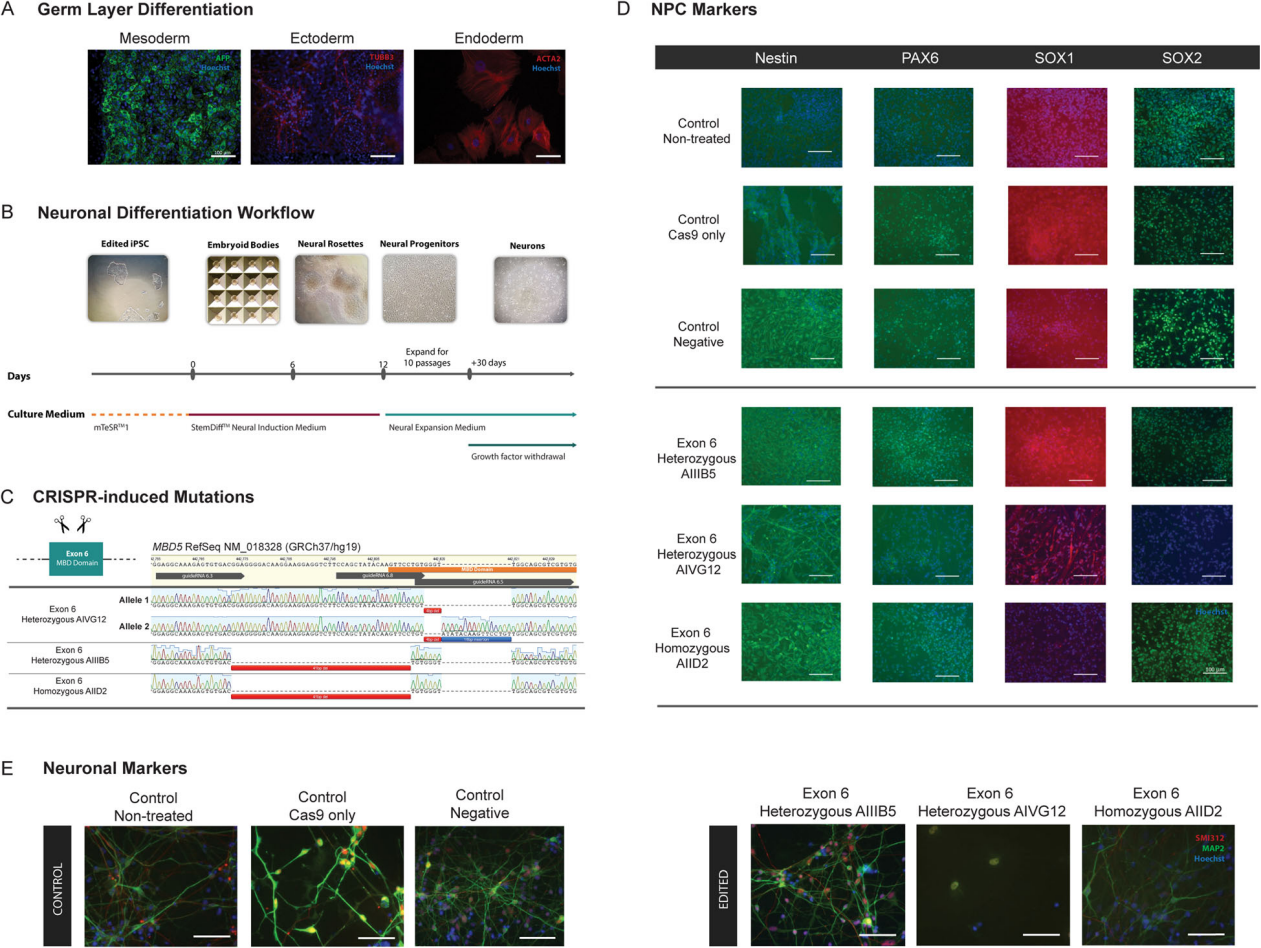
CRISPR cell line characterization and differentiation. **a** 8330-8 iPSC were able to generate all three germ layers. **b** Differentiation workflow of iPSC into NPCs and mature neurons. **c** CRISPR-induced mutations in the first coding of *MBD5*, exon 6. **d** NPC. **e** Neuron staining of CRISPR-edited and control cell lines, using lineage-specific markers

**Fig. 5:**
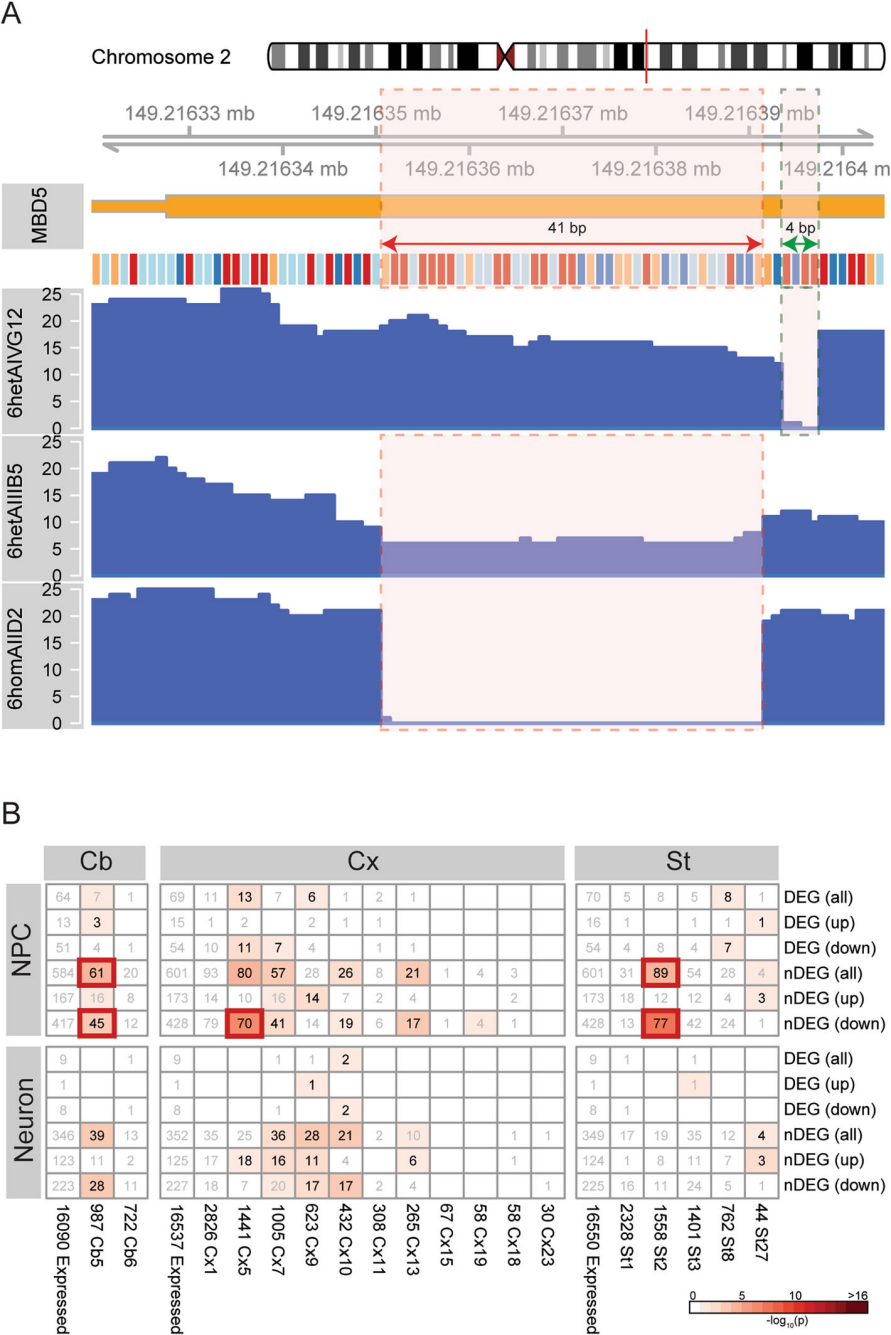
Expression profiling of CRISPR/Cas9 edited cell lines. **a** RNA-seq read coverage of the exon 6 in CRISPR/Cas9-edited NPCs. The 41 bp deletion is highlighted in red, and 4 bp insertion is highlighted in green. **b** Enrichments of nDEGs from cell lines in mouse co-expression modules. The color indicates the significance (−log_10_(*p*)) of Fisher’s enrichment test, and the number shows the number of common genes. Red squares highlight the significant enrichments after adjustment for multiple hypothesis testing

With the goal of validating whether those genes and biological processes that showed dysregulation in mouse brain regions also appeared to be altered by MBD5 reduction in human neuronal cells, we generated CRISPR/Cas9-edited iPSC lines for assessment of NPCs and neurons with *MBD5* mutation. We specifically created a heterozygous exon 6 coding deletion, affecting the MBD domain on one allele to model haploinsufficiency. We also generated a line homozygous for the same mutation as the heterozygote as well as a compound heterozygous line harboring a different mutation on each allele (Fig. 4c), resulting in three cell lines with impaired MBD5 transcript (Fig. 5a) and three control cell lines. The gene expression profiles of these cell lines show that they cluster according to their differentiation stage, with the exception of the compound cell line 6AIVG12 that clustered together with the NPCs, in accordance to our previous observations (Supplementary Figure 4). Like the mouse brain region analysis, in comparing human NPCs and neurons there was statistically significant enrichment for overlap between nDEGs that was more prominent for downregulated genes (Supplementary Figure 5: upregulation enrichment *p* value = 3.5 × 10^−10^, downregulation enrichment *p* value = 1.1 × 10^−21^, Supplementary Table 7).

Given the limited power to detect differential expression for independent genome-wide conclusions in the small cell line panel, we focused on testing overlap with the mouse data by comparing nDEGs from both mouse brain and cell lines. Again, there was limited but significant overlap that was more prominent among downregulated genes (Fig. 2c), with particularly the nDEGs from NPCs being enriched in specific modules from the three mouse brain regions (Fig. 5b). We again performed a meta-analysis of *p* values of 848 genes with common direction of regulation (349—up and 499—down) in the mouse brain regions and the cell lines using Fisher’s method to identify any genes similarly dysregulated in all 5 RNA sources. Although this revealed 38 genes to be significant, 36 down regulated and 2 upregulated (Supplementary Figure 6A), there was no gene other than *MBD5/Mbd5* that was nominally significant in all cases, again supporting largely different effects of MBD5 reduction in different cellular contexts.

This conclusion was reinforced by our analysis of an independent human cell line dataset from Gigek et al. [63]. While we used iPSCs reprogrammed from a primary culture of skin fibroblasts and differentiated these iPSCs into NPCs and neurons, Gigek et al. used a commercial human neural progenitor cell line (ReNcell® VM) that was derived from a particular brain region, the ventral mesencephalon of the human fetal brain, and was immortalized by myc transduction, in contrast to our reprogrammed untransformed cell lines. Gigek et al. performed RNA sequencing on two NSC cell lines using *MBD5* shRNA knock-downs, in contrast to our CRISPR experiment that creates permanent modifications to the DNA sequence of the cells. The Gigek et al. dataset comprised 556 DEGs at FDR < 0.01. We tested for enrichment of shared DEGs across the human cell line nDEGs from our study with downregulated and upregulated DEGs from the Gigek et al. study and found statistically significant overlap in the same direction for downregulated DEGs in NPCs and neurons, but not for upregulated DEGs (Supplementary Figure 6B). No significant overlap in the same direction was detected between the Gigek et al. cell lines and any of the mouse brain regions. This finding with an independent neuronal cell line of different origin and mechanism of MBD5 reduction supports the view that the consequences of lowering MBD5 are dependent on the specific cell type and cell state being analyzed, rather than affecting one or more pathways consistently across all cell types. These datasets used and/or analyzed in this study are available in the gene expression omnibus (GEO) with accession numbers GSE144277 (mouse) and GSE144279 (cell lines).

## Discussion

Disruption of *MBD5* has been reproducibly shown to be associated with ASD and NDD across a number of independent studies using varying designs and mutational classes. It is one of many genes involved in regulatory pathways associated with these disorders. However, despite the substantial evidence that alteration of this gene adversely affects neurodevelopment, little is known about the biological processes that are altered as a consequence of haploinsufficiency of *MBD5.* The mouse brain regions were selected based on their potential relevance for ASD and the presence of *Mbd5* expression. *Mbd5* expression in *Mbd5*^+/GT^ mice was observed throughout the brain, most prominently in the cortex, cerebellum and striatum [19]. The cortex controls many of the executive functions of the brain, including higher-order cognitive processes, such as emotions, social behavior, learning, and communication, that are impaired in ASD patients. The main observations in ASD brains include abnormal cortical growth patterns [64], abnormalities in cortical thickness and disorganization of neurons across the cortical layers and their connections to other regions of the brain [65, 66]. Abnormalities in the cerebellum have also been reported in several clinical studies of individuals with ASD, including increased cerebellar activation during a motor task [67] and a reduction in cerebellar Purkinje cells, the most often reported post-mortem finding [68, 69]. Indeed, neonatal cerebellar damage confers a large risk (up to 40%) for developing ASD later in life [69, 70], which highlights the relevance of this brain region in ASD. According to the GTEx database of human postmortem tissues (http://www.gtexportal.org/), *MBD5* shows the highest mRNA expression levels in the cerebellum. Regarding the striatum, several ASD risk genes have been shown to be important for striatal function, including *FOXP1* [71, 72] and *FOXP2* [73] and *SHANK3* [74].

Here, gene expression analyses of three brain regions from *Mbd5*^+/GT^ mice showed subtle changes, with cortex as the region most affected and lesser overall effects in cerebellum and striatum, even though all showed a reduction of *Mbd5* expression, demonstrating apparent context-dependent consequences of *Mbd5* deficiency. The difference in *Mbd5* effect in these brain regions may be explained in part by the substantial inherent difference in expression profiles that these regions display. These differences can be easily observed in principal component analysis of gene expression in the mouse brain (Supplementary Figure 7), where brain region, not *Mbd5* genotype, is the primary component of the variability in gene expression, contributing as much as 79% of the overall variation. However, even when there is evidence for similar processes in different brain regions, such as the related gene co-expression modules observed in cortex and striatum, the effect of *Mdb5* reduction varies by region: cortex nDEGs were enriched in some corresponding cortex and striatal modules but striatal nDEGs were not enriched in those same modules and vice versa. Further, some modules were enriched for cortical and striatal nDEGs dysregulated in opposite directions. The concept that the subtle effects of reduced MBD5 expression do not alter a fixed set of pathways across all tissues is consistent the dysregulation in the same direction being shared by only a handful of nDEGs across the three brain regions, and none being shared by these and the iPSC-derived NPCs and neurons. Indeed, the view that MBD5 deficiency can have quite different effects depending on cell type and cell state is evident in the minimal significant overlap between gene expression differences in our human cell lines and those from a previously published immortalized fetal NPC with MDB5 shRNA knockdown [63], and the lack of significant overlap of the latter DEGs with the mouse brain results. Consequently, our results implicate a role for MBD5 that is adapted to the regulatory needs of the cell type and tissue in question, with subtle quantitative effects of MBD5 reduction that are not highly predictive of its effects in other cell types/stages. Defining which consequences of *MBD5* haploinsufficiency are critical for causing NDD will therefore likely require detailed analysis across different cell types and brain regions through early development, including most likely the need for single-cell gene expression analyses.

With respect to cortex in particular, one surprising candidate that is worthy of further investigation is the ciliary function, implicated by the WGCNA module DEG enrichments. Cortical neuronal progenitors and developing neurons have a primary cilium—a microtubule-based, slender, antenna-like projection—that is an essential integrator and conveyor of signal transduction [75]. Indeed, primary cilia sense and transduce many extracellular signals to influence a wide variety of processes, such as cell proliferation and polarity, developmental processes and neuronal growth [76]. For example, studies have shown that primary cilia are important in the regulation of neuronal progenitor cell proliferation and the generation of neurons both in the cerebral cortex [77] as well as in orchestrating the coordinated migration and placement of postmitotic interneurons in the developing cerebral cortex [78, 79]. NDDs such as autism and schizophrenia are associated with human ciliopathies [80–82] and this suggests that impaired ciliary function can hinder the development of neural circuitry and activity, leading to significant functional deficits. In fact, several well-established ASD genes are directly involved in ciliary biology such as *DISC1*, *CTNNB1* or their knockdowns result in cilia loss (e.g., *KATNAL2*, *NRXN1*, *FOXP1*, and *CHD7*) [83]. Our detection in 8-week old brain of corresponding modules in both cortex and striatum that show enrichment for GO terms related to cilium and of upregulated cortex nDEGs enriched in these modules suggests the possibility that disruption of a cilium-related process may also occur earlier in cortical development due to MBD5 deficiency.

In summary, our gene expression analysis of mouse and human cell models of *MBD5* haploinsufficiency did not reveal a common disrupted pathway, but rather pointed to subtle effects critically dependent on cell context, complicating the search for the precise mechanism(s) by which this genetic lesion leads to NDD but providing a resource for its further investigation.

## Limitations

The transcriptional changes associated with reduced *Mbd5* expression in this study are modest by comparison to what has been observed from studies of other ASD-associated genes in mouse and neuronal models, such as *CHD8* [36, 84], and the profound neurodevelopmental changes associated with its disruption in humans. There are several factors that may influence this result, including the limitation of using mouse and neuronal models to mimic the pathogenic processes associated *MBD5* alterations in NDD cases, the sensitivity of the assays to detect the spectrum of developmental changes that occur, and the unknown periods of developmental timing at which *MBD5* exerts its greatest effects. While we explored multiple mouse brain regions and two different neuronal lineages, and we broadened our analyses to incorporate nominally significant DEGs, these analyses do not overcome the limitations to the interpretation of the pathogenic mechanisms that occur in humans. The ultimate conclusion that reduced expression of MBD5 has effects that are highly context-dependent dictates that future evaluation of *Mbd5* haploinsufficiency in NDD will require detailed cell-specific analysis across early development.

## Conclusions

Our study begins to explore the transcriptional consequences of reduced *Mbd5* expression in mouse brain regions, its validation in a human neuronal model, and comparison with a previously published human cell model. Our findings point to reduced levels of MBD5 having modest effects on gene expression that are highly dependent on cellular context. The highest number of gene expression changes occurred in the cortex, a brain region important in NDD, and point to the possibility of perturbation to normal ciliary function due to *MBD5* haploinsufficiency. However, the wide variation in effects across all models suggests that *MBD5* disruption does not alter a critical set of pathways across brain regions, but rather has different effects in different cell types and regions, complicating interpretation of which disruptions may contribute to neurodevelopmental defects. Future studies combining earlier stages of development and advanced models such as brain organoids with single-cell RNAseq will be essential to tease apart specific developmental timings and cell-specific profiles and further explore the exact mechanism by which this gene regulates crucial pathways during brain development that, when gone awry, contribute to NDDs.

## Supplementary information

**Additional file 1: Table S1.** Information of mouse samples collected. **Table S2.** Guide RNAs used for *MBD5* gene editing. **Table S3.** Complete list of all expressed genes tested for differential expression in each mouse brain region, corresponding statistics and co-expression module assignment. **Table S4.** Genes that showed regulation in the same direction at nominal significance in at least one mouse brain region and at least one cell line. **Table S5.** Description of the gene lists with relevance to neuronal development and function and corresponding publications. **Table S6.** Complete delineation of genes comprising the gene lists in Supplementary Table 5. **Table S7.** Complete list of all expressed genes tested for differential expression in NPCs and neurons and corresponding statistics.

**Additional file 2: Figure S1.** Enrichments of differentially expressed genes in gene sets with relevance to neurodevelopment and neuronal function. The description of gene lists and corresponding publications is provided in Supplementary Tables 5 & 6. The color represents -log10(p-value). **Figure S2.** Enrichments of co-expression modules with evidence of Mbd5 knock-down relevance in gene sets with relevance to neurodevelopment and neuronal function. Only sets with significant enrichments are shown. The description of gene lists and corresponding publications is provided in Supplementary Tables 5 & 6. The color represents -log10(p-value). **Figure S3.** Protein-protein interaction network of genes from co-expression module Cx15 from String-db database. The nodes filled with red represent the genes that belong to GO “cilium”. Nodes circled in red are differentially expressed in cortex at nominal p-value <0.05. The boxplot shows the mean expression of the genes in module Cx15 as normalized log10-transformed counts. **Figure S4.** Heatmap of gene expression of cell-type specific markers as normalized log-transformed scaled counts. The values are scaled by row. **Figure S5.** Differential expression analysis of cell lines and overlaps with mouse brain regions. A-B - Volcano plots of differential expression tests for NPCs (A) and Neurons (B). X-axis shows estimated log2 fold change and y-axis shows -log10(FDR). Horizontal grey dashed line shows -log10(0.05), marking the significance cut-off for FDR. Vertical grey dashed line shows the log2 fold change = 0. Red points show the genes that have FDR < 0.05 and absolute log2 fold change less or equal to 1, green points show the genes with FDR < 0.05 and absolute log2 fold change greater than 1. C - Table of number of differentially expressed genes in NPCs and Neurons at FDR < 0.05 and nominal p < 0.05. D - Overlap of nominal differentially expressed genes in cell lines and mice. Genes that are expressed in all 5 comparisons (NPCs, neurons, mouse cerebellum, mouse cortex, mouse striatum) were considered as background for enrichment tests. The number inside the cell shows number of background genes in corresponding overlap, and the color of the cell shows the -log10(p) from Fisher's test for overrepresentation. **Figure S6.** Meta analysis of cell lines using Fisher’s method and comparison of nDEGs with Gigek et al. A - Genes with FDR < 0.05 in meta-analysis on all mouse regions and cell lines. The heatmap shows the direction and significance of each gene in the corresponding cell type/brain region. B –Enrichment of DEGs identified in Gigek et al. among nDEGs from mouse brain regions, and human NPCs and neurons. The color indicates -log10(p) of Fisher’s enrichment test between two sets, and the number shows the number of genes in common. **Figure S7.** Principal Component Analysis of mouse brain regions. This shows that the primary component of the variability in gene expression is brain region, contributing as much as 79% to overall variation.

## Data Availability

The datasets used and/or analyzed during the current study were submitted to gene expression omnibus (GEO) with accession numbers GSE144277 (mouse) and GSE144279 (cell lines) and are available from the corresponding author upon request.

## Abbreviations

ASD: Autism spectrum disorder
CRISPR: Clustered regularly interspaced short palindromic repeats
DEG: Differentially expressed genes
FDR: False discovery rate
GO: Gene ontology
GPCR: G-protein-coupled receptors
GSEA: Gene set enrichment analysis
KEGG: Kyoto encyclopedia of genes and genomes
MBD: Methyl-binding domain
NDD: Neurodevelopmental disorders
nDEG: Nominally differentially expressed genes
PWWP: Proline and tryptophan-rich
WGCNA: Weighted gene co-expression network analysis
