# Supplementary figures and images for: Transcriptional consequences of MBD5 disruption in mouse brain and CRISPR-derived neurons
Source: Mol Autism. 2020 Jun 5;11:45. doi: 10.1186/s13229-020-00354-1 (PMC7275313; doi:10.1186/s13229-020-00354-1)

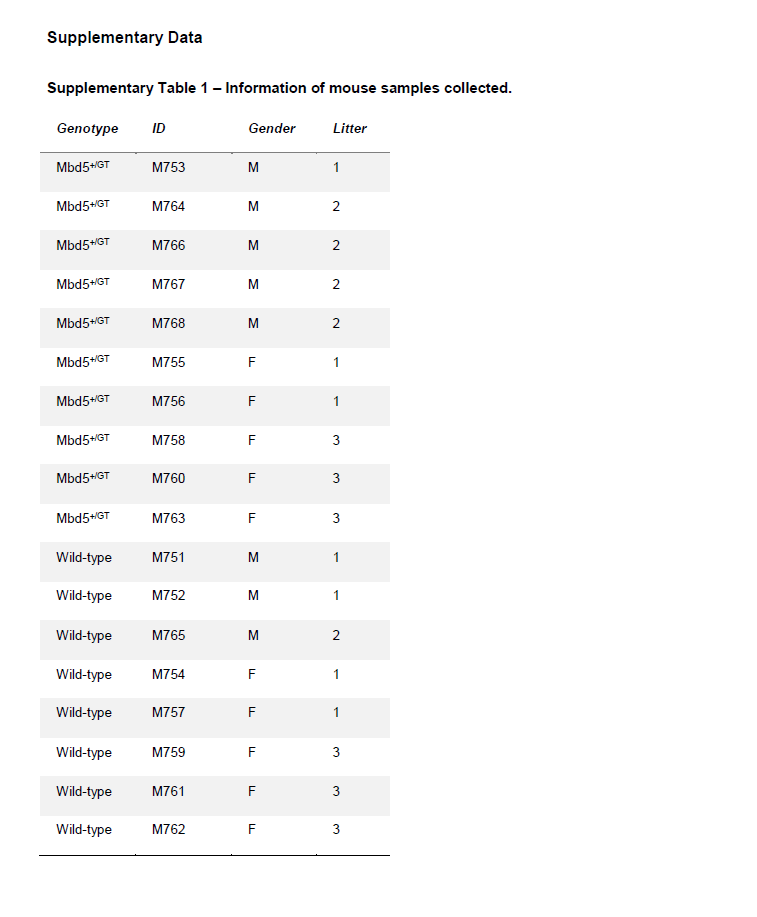

Supplement: Supplementary file 1 — Additional file 1: Table S1. Information of mouse samples collected. Table S2. Guide RNAs used for MBD5 gene editing. Table S3. Complete list of all expressed genes tested for differential expression in each mouse brain region, corresponding statistics and co-expression module assignment. Table S4. Genes that showed regulation in the same direction at nominal significance in at least one mouse brain region and at least one cell line. Table S5. Description of the gene lists with relevance to neuronal development and function and corresponding publications. Table S6. Complete delineation of genes comprising the gene lists in Supplementary Table 5. Table S7. Complete list of all expressed genes tested for differential expression in NPCs and neurons and corresponding statistics. [file 13229_2020_354_MOESM1_ESM.zip › supplementary table 1.PNG]

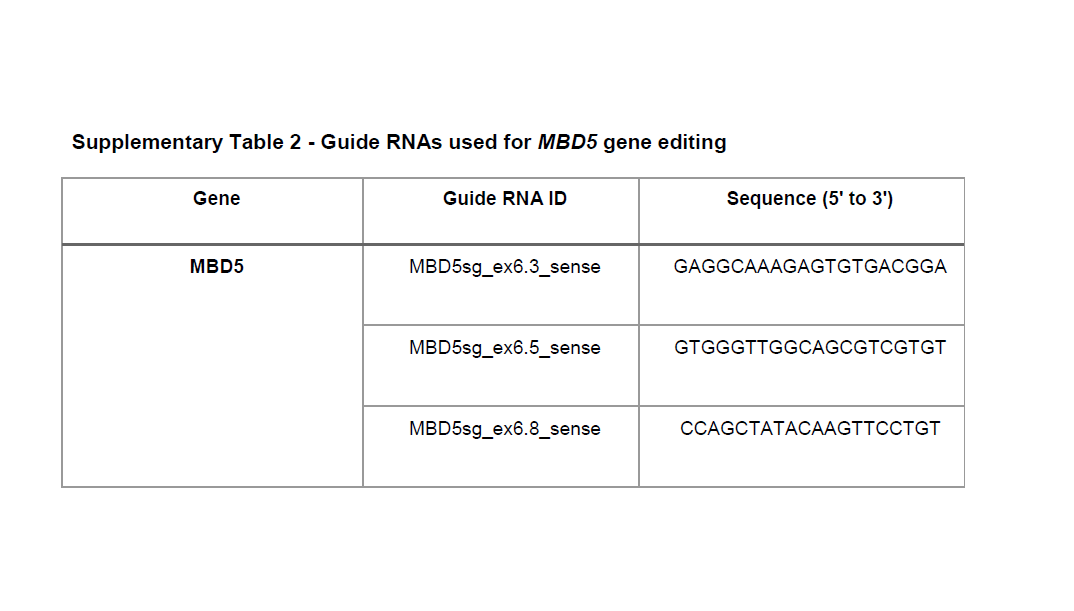

Supplement: Supplementary file 1 — Additional file 1: Table S1. Information of mouse samples collected. Table S2. Guide RNAs used for MBD5 gene editing. Table S3. Complete list of all expressed genes tested for differential expression in each mouse brain region, corresponding statistics and co-expression module assignment. Table S4. Genes that showed regulation in the same direction at nominal significance in at least one mouse brain region and at least one cell line. Table S5. Description of the gene lists with relevance to neuronal development and function and corresponding publications. Table S6. Complete delineation of genes comprising the gene lists in Supplementary Table 5. Table S7. Complete list of all expressed genes tested for differential expression in NPCs and neurons and corresponding statistics. [file 13229_2020_354_MOESM1_ESM.zip › Supplementary table 2.PNG]

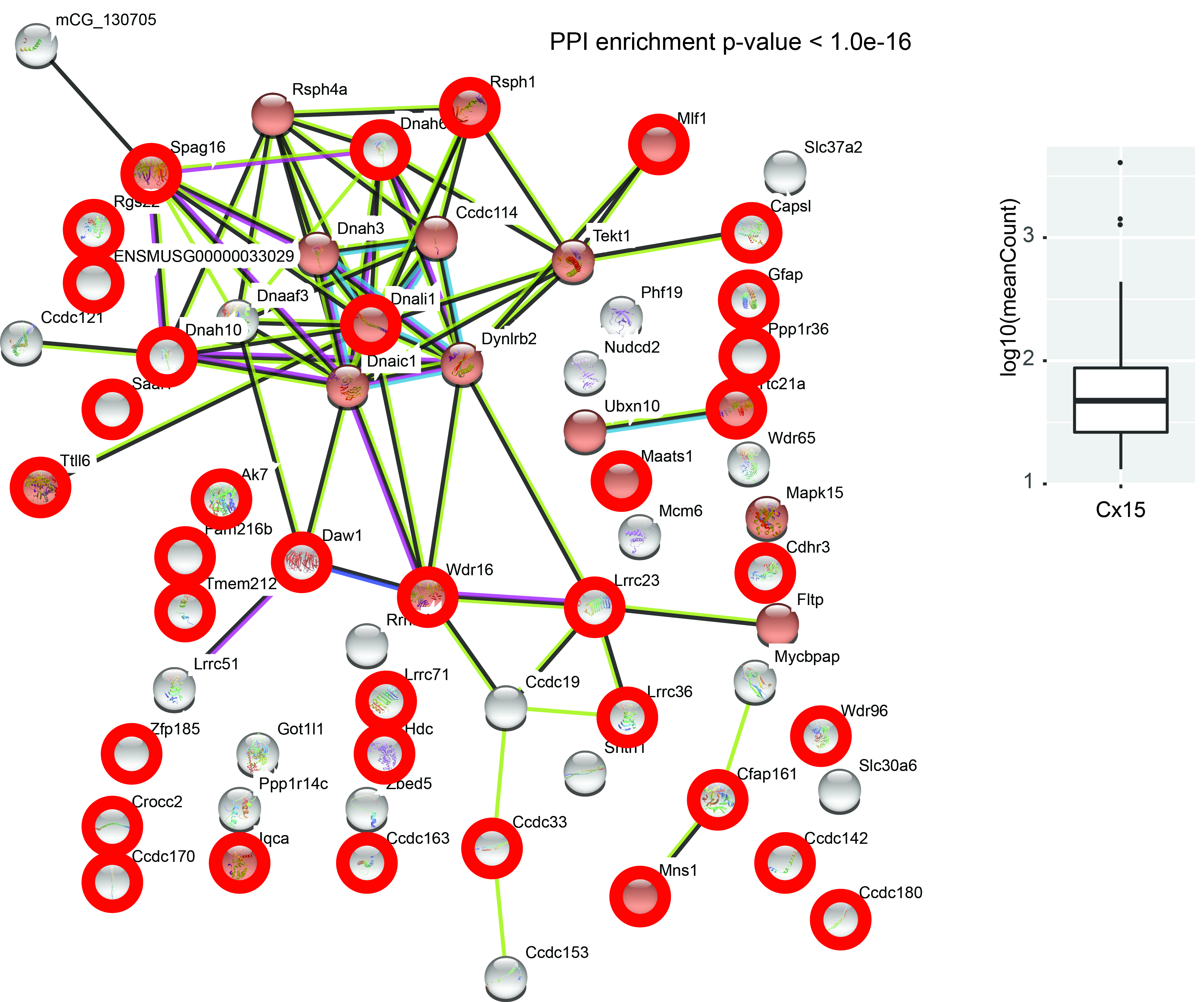

Supplement: Supplementary file 2 — Additional file 2: Figure S1. Enrichments of differentially expressed genes in gene sets with relevance to neurodevelopment and neuronal function. The description of gene lists and corresponding publications is provided in Supplementary Tables 5 & 6. The color represents -log10(p-value). Figure S2. Enrichments of co-expression modules with evidence of Mbd5 knock-down relevance in gene sets with relevance to neurodevelopment and neuronal function. Only sets with significant enrichments are shown. The description of gene lists and corresponding publications is provided in Supplementary Tables 5 & 6. The color represents -log10(p-value). Figure S3. Protein-protein interaction network of genes from co-expression module Cx15 from String-db database. The nodes filled with red represent the genes that belong to GO “cilium”. Nodes circled in red are differentially expressed in cortex at nominal p-value <0.05. The boxplot shows the mean expression of the genes in module Cx15 as normalized log10-transformed counts. Figure S4. Heatmap of gene expression of cell-type specific markers as normalized log-transformed scaled counts. The values are scaled by row. Figure S5. Differential expression analysis of cell lines and overlaps with mouse brain regions. A-B - Volcano plots of differential expression tests for NPCs (A) and Neurons (B). X-axis shows estimated log2 fold change and y-axis shows -log10(FDR). Horizontal grey dashed line shows -log10(0.05), marking the significance cut-off for FDR. Vertical grey dashed line shows the log2 fold change = 0. Red points show the genes that have FDR < 0.05 and absolute log2 fold change less or equal to 1, green points show the genes with FDR < 0.05 and absolute log2 fold change greater than 1. C - Table of number of differentially expressed genes in NPCs and Neurons at FDR < 0.05 and nominal p < 0.05. D - Overlap of nominal differentially expressed genes in cell lines and mice. Genes that are expressed in all 5 comparisons (NPCs, neurons, m [file 13229_2020_354_MOESM2_ESM.zip › Supplementary_Figure_3.tif]

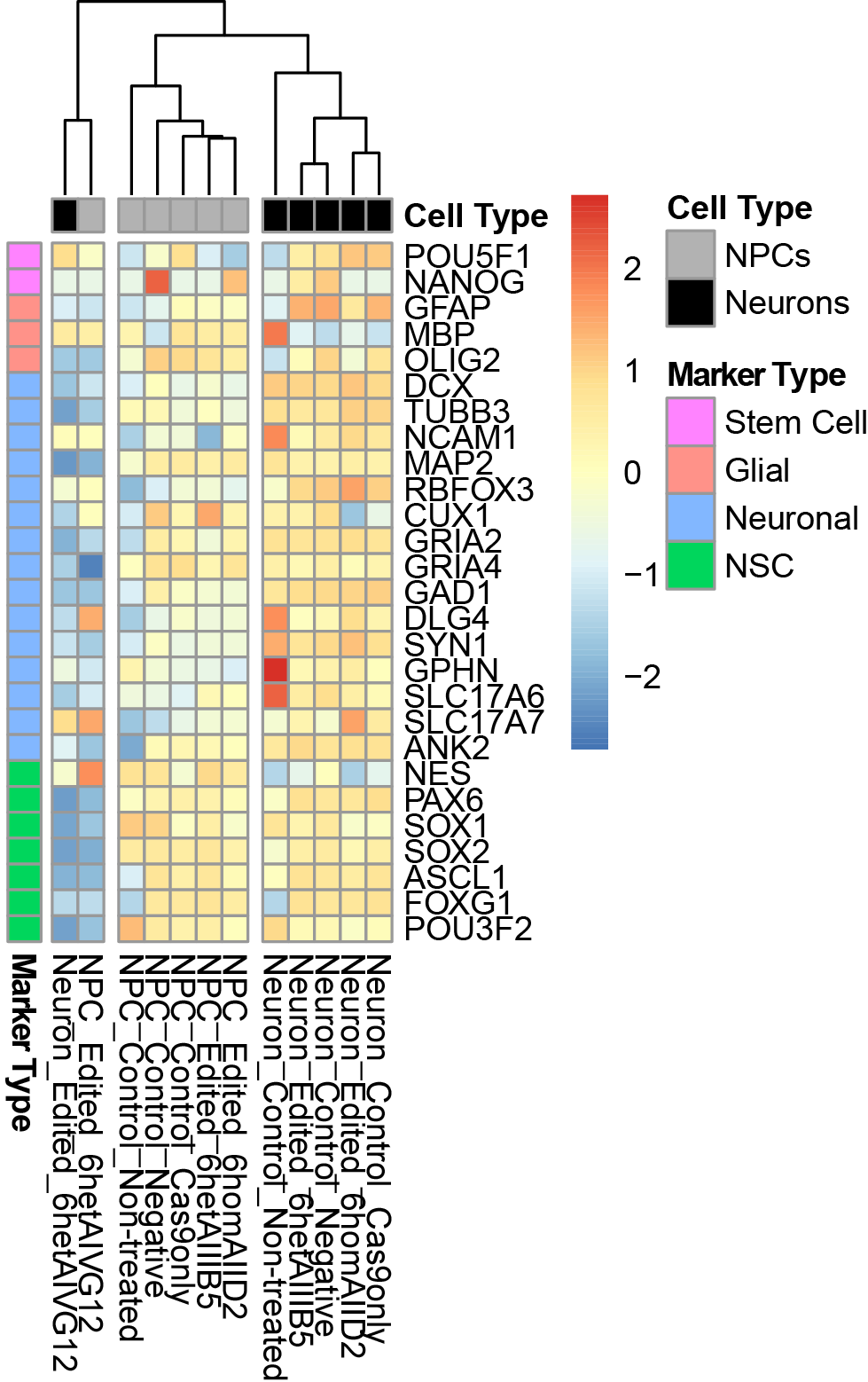

Supplement: Supplementary file 2 — Additional file 2: Figure S1. Enrichments of differentially expressed genes in gene sets with relevance to neurodevelopment and neuronal function. The description of gene lists and corresponding publications is provided in Supplementary Tables 5 & 6. The color represents -log10(p-value). Figure S2. Enrichments of co-expression modules with evidence of Mbd5 knock-down relevance in gene sets with relevance to neurodevelopment and neuronal function. Only sets with significant enrichments are shown. The description of gene lists and corresponding publications is provided in Supplementary Tables 5 & 6. The color represents -log10(p-value). Figure S3. Protein-protein interaction network of genes from co-expression module Cx15 from String-db database. The nodes filled with red represent the genes that belong to GO “cilium”. Nodes circled in red are differentially expressed in cortex at nominal p-value <0.05. The boxplot shows the mean expression of the genes in module Cx15 as normalized log10-transformed counts. Figure S4. Heatmap of gene expression of cell-type specific markers as normalized log-transformed scaled counts. The values are scaled by row. Figure S5. Differential expression analysis of cell lines and overlaps with mouse brain regions. A-B - Volcano plots of differential expression tests for NPCs (A) and Neurons (B). X-axis shows estimated log2 fold change and y-axis shows -log10(FDR). Horizontal grey dashed line shows -log10(0.05), marking the significance cut-off for FDR. Vertical grey dashed line shows the log2 fold change = 0. Red points show the genes that have FDR < 0.05 and absolute log2 fold change less or equal to 1, green points show the genes with FDR < 0.05 and absolute log2 fold change greater than 1. C - Table of number of differentially expressed genes in NPCs and Neurons at FDR < 0.05 and nominal p < 0.05. D - Overlap of nominal differentially expressed genes in cell lines and mice. Genes that are expressed in all 5 comparisons (NPCs, neurons, m [file 13229_2020_354_MOESM2_ESM.zip › Supplementary_Figure_4.png]

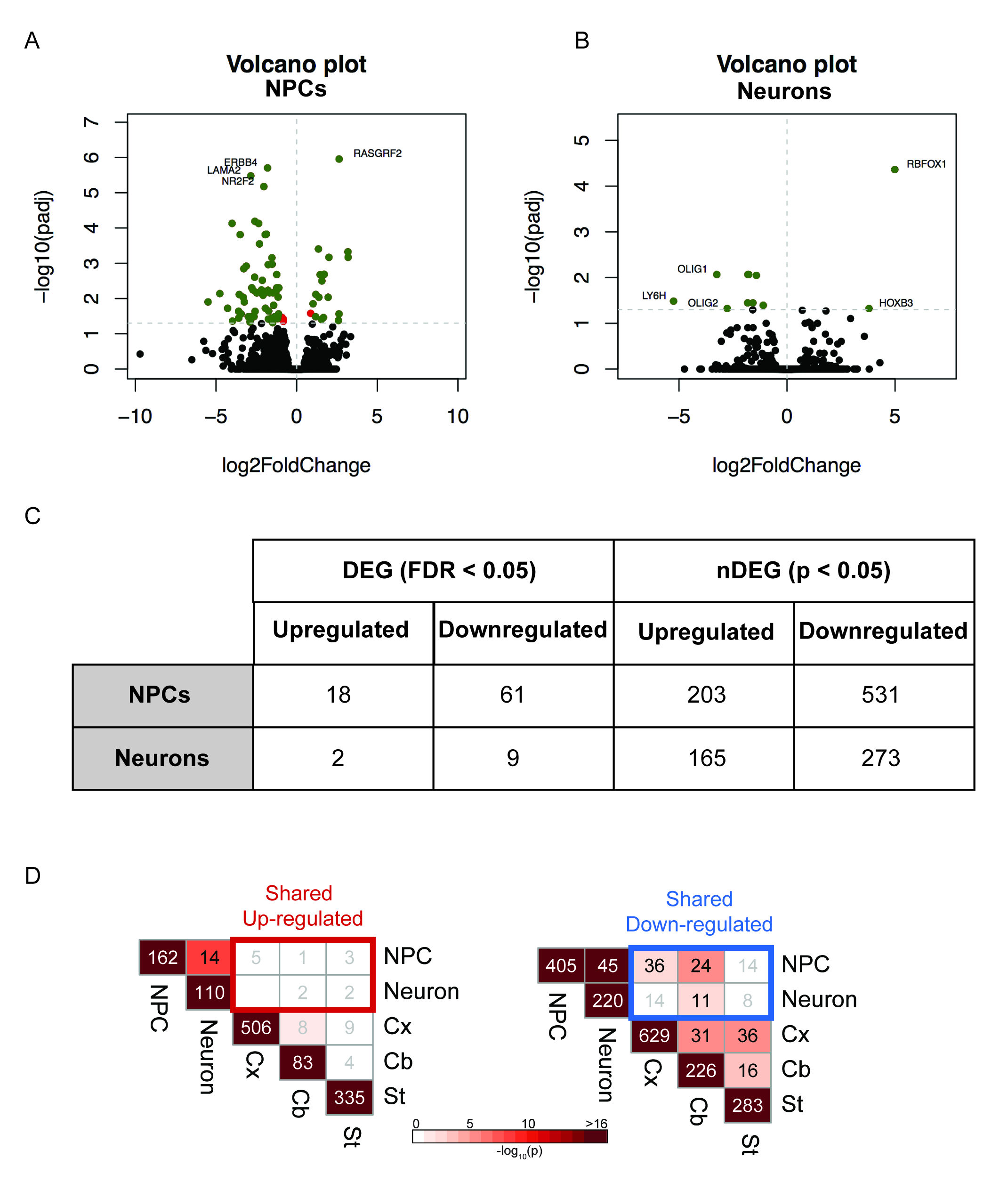

Supplement: Supplementary file 2 — Additional file 2: Figure S1. Enrichments of differentially expressed genes in gene sets with relevance to neurodevelopment and neuronal function. The description of gene lists and corresponding publications is provided in Supplementary Tables 5 & 6. The color represents -log10(p-value). Figure S2. Enrichments of co-expression modules with evidence of Mbd5 knock-down relevance in gene sets with relevance to neurodevelopment and neuronal function. Only sets with significant enrichments are shown. The description of gene lists and corresponding publications is provided in Supplementary Tables 5 & 6. The color represents -log10(p-value). Figure S3. Protein-protein interaction network of genes from co-expression module Cx15 from String-db database. The nodes filled with red represent the genes that belong to GO “cilium”. Nodes circled in red are differentially expressed in cortex at nominal p-value <0.05. The boxplot shows the mean expression of the genes in module Cx15 as normalized log10-transformed counts. Figure S4. Heatmap of gene expression of cell-type specific markers as normalized log-transformed scaled counts. The values are scaled by row. Figure S5. Differential expression analysis of cell lines and overlaps with mouse brain regions. A-B - Volcano plots of differential expression tests for NPCs (A) and Neurons (B). X-axis shows estimated log2 fold change and y-axis shows -log10(FDR). Horizontal grey dashed line shows -log10(0.05), marking the significance cut-off for FDR. Vertical grey dashed line shows the log2 fold change = 0. Red points show the genes that have FDR < 0.05 and absolute log2 fold change less or equal to 1, green points show the genes with FDR < 0.05 and absolute log2 fold change greater than 1. C - Table of number of differentially expressed genes in NPCs and Neurons at FDR < 0.05 and nominal p < 0.05. D - Overlap of nominal differentially expressed genes in cell lines and mice. Genes that are expressed in all 5 comparisons (NPCs, neurons, m [file 13229_2020_354_MOESM2_ESM.zip › Supplementary_Figure_5.tif]

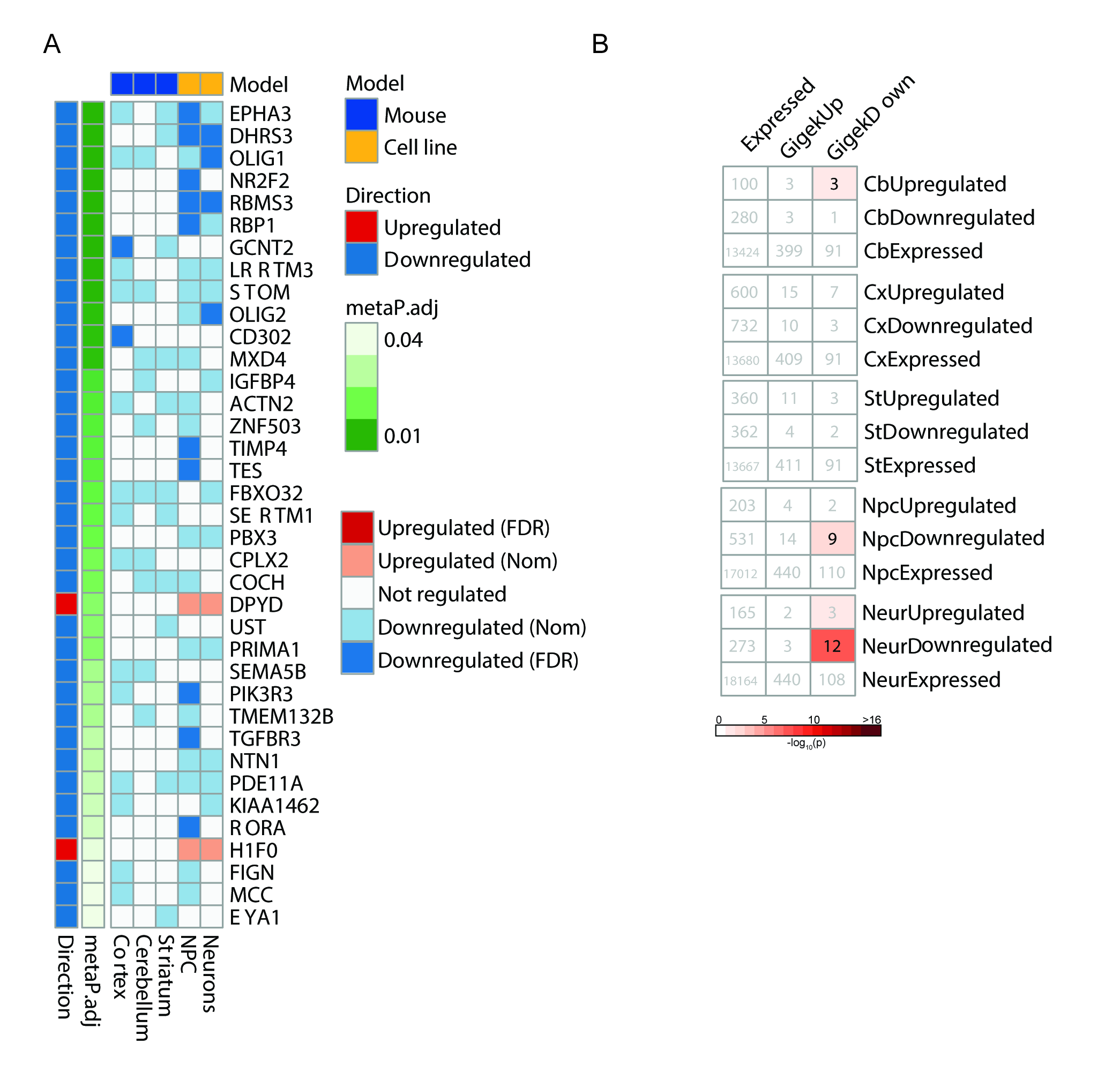

Supplement: Supplementary file 2 — Additional file 2: Figure S1. Enrichments of differentially expressed genes in gene sets with relevance to neurodevelopment and neuronal function. The description of gene lists and corresponding publications is provided in Supplementary Tables 5 & 6. The color represents -log10(p-value). Figure S2. Enrichments of co-expression modules with evidence of Mbd5 knock-down relevance in gene sets with relevance to neurodevelopment and neuronal function. Only sets with significant enrichments are shown. The description of gene lists and corresponding publications is provided in Supplementary Tables 5 & 6. The color represents -log10(p-value). Figure S3. Protein-protein interaction network of genes from co-expression module Cx15 from String-db database. The nodes filled with red represent the genes that belong to GO “cilium”. Nodes circled in red are differentially expressed in cortex at nominal p-value <0.05. The boxplot shows the mean expression of the genes in module Cx15 as normalized log10-transformed counts. Figure S4. Heatmap of gene expression of cell-type specific markers as normalized log-transformed scaled counts. The values are scaled by row. Figure S5. Differential expression analysis of cell lines and overlaps with mouse brain regions. A-B - Volcano plots of differential expression tests for NPCs (A) and Neurons (B). X-axis shows estimated log2 fold change and y-axis shows -log10(FDR). Horizontal grey dashed line shows -log10(0.05), marking the significance cut-off for FDR. Vertical grey dashed line shows the log2 fold change = 0. Red points show the genes that have FDR < 0.05 and absolute log2 fold change less or equal to 1, green points show the genes with FDR < 0.05 and absolute log2 fold change greater than 1. C - Table of number of differentially expressed genes in NPCs and Neurons at FDR < 0.05 and nominal p < 0.05. D - Overlap of nominal differentially expressed genes in cell lines and mice. Genes that are expressed in all 5 comparisons (NPCs, neurons, m [file 13229_2020_354_MOESM2_ESM.zip › Supplementary_Figure_6.tif]

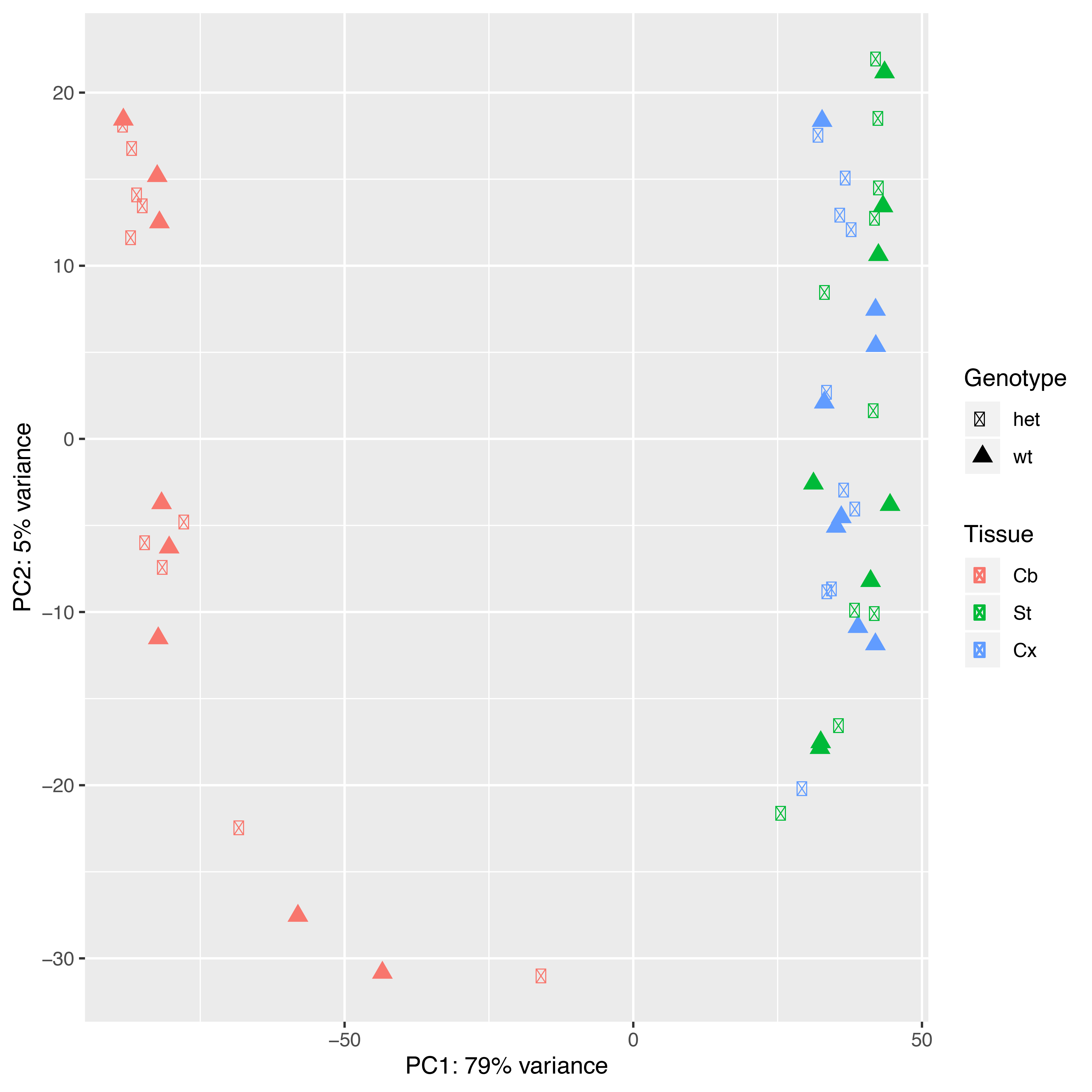

Supplement: Supplementary file 2 — Additional file 2: Figure S1. Enrichments of differentially expressed genes in gene sets with relevance to neurodevelopment and neuronal function. The description of gene lists and corresponding publications is provided in Supplementary Tables 5 & 6. The color represents -log10(p-value). Figure S2. Enrichments of co-expression modules with evidence of Mbd5 knock-down relevance in gene sets with relevance to neurodevelopment and neuronal function. Only sets with significant enrichments are shown. The description of gene lists and corresponding publications is provided in Supplementary Tables 5 & 6. The color represents -log10(p-value). Figure S3. Protein-protein interaction network of genes from co-expression module Cx15 from String-db database. The nodes filled with red represent the genes that belong to GO “cilium”. Nodes circled in red are differentially expressed in cortex at nominal p-value <0.05. The boxplot shows the mean expression of the genes in module Cx15 as normalized log10-transformed counts. Figure S4. Heatmap of gene expression of cell-type specific markers as normalized log-transformed scaled counts. The values are scaled by row. Figure S5. Differential expression analysis of cell lines and overlaps with mouse brain regions. A-B - Volcano plots of differential expression tests for NPCs (A) and Neurons (B). X-axis shows estimated log2 fold change and y-axis shows -log10(FDR). Horizontal grey dashed line shows -log10(0.05), marking the significance cut-off for FDR. Vertical grey dashed line shows the log2 fold change = 0. Red points show the genes that have FDR < 0.05 and absolute log2 fold change less or equal to 1, green points show the genes with FDR < 0.05 and absolute log2 fold change greater than 1. C - Table of number of differentially expressed genes in NPCs and Neurons at FDR < 0.05 and nominal p < 0.05. D - Overlap of nominal differentially expressed genes in cell lines and mice. Genes that are expressed in all 5 comparisons (NPCs, neurons, m [file 13229_2020_354_MOESM2_ESM.zip › Supplementary_Figure_7.tif]
